# Distinct Neonatal Brain Anatomy Is Associated With Cross‐Disorder Genetic Risk for Psychiatric Disorders

**DOI:** 10.1002/hbm.70532

**Published:** 2026-04-19

**Authors:** Xixi Dang, Ruiqi Su, Dan Wu, Mingyang Li

**Affiliations:** ^1^ Department of Psychology Hangzhou Normal University Hangzhou China; ^2^ Zhejiang Philosophy and Social Science Laboratory for Research in Early Development and Childcare Hangzhou Normal University Hangzhou China; ^3^ Key Laboratory for Biomedical Engineering of Ministry of Education, Department of Biomedical Engineering, College of Biomedical Engineering & Instrument Science Zhejiang University Hangzhou China; ^4^ Children's Hospital Zhejiang University School of Medicine, National Clinical Research Center for Child Health Hangzhou China

**Keywords:** brain structure, cross‐disorder factor, neonatal brain, neurodevelopment, polygenic risk score

## Abstract

Psychiatric disorders share a complex polygenic architecture, yet how this genetic liability relates to early brain development remains unclear. This study investigated the associations between cross‐disorder polygenic risk and neonatal brain anatomy. We derived three latent psychiatric factors using GenomicSEM, reflecting shared genetic liability among related conditions: A neurodevelopmental factor, a compulsive factor, and a mood‐psychosis factor. We then calculated respective polygenic risk scores (PRS) in 336 neonates from the Developing Human Connectome Project. We found that cross‐disorder PRSs (neurodevelopmental and mood‐psychosis factors) showed significantly broader associations with neonatal brain volumes than disorder‐specific PRSs. These associations were highly robust, as confirmed through validation analyses using updated GWAS data and an alternative PRS method (PRS‐CS). These cross‐disorder PRSs were strongly correlated with smaller global brain size. After accounting for this global effect, associations with a subset of brain regions remained detectable. In exploratory analyses, the neurodevelopmental factor was reproducibly linked to heightened alertness at 18 months. Our results reveal that shared genetic risk for psychiatric disorders manifests as both global and regionally specific variations in brain anatomy at birth, highlighting the value of cross‐disorder genetic models for elucidating early neurodevelopmental vulnerability.

## Key Points

- Cross‐disorder polygenic risk scores (PRS) demonstrate broader associations with neonatal brain structure than disorder‐specific scores.
- The shared genetic risk is robustly associated with smaller global brain size, with suggestive, more limited regional effects persisting after accounting for overall brain volume.
- The neurodevelopmental PRS is linked to alertness as early as 18 months of age.

## Graphical abstract

This study shows that shared genetic risks for psychiatric disorders are already linked to specific variations in newborn brain structure. This uncovers the initial brain manifestations of genetic vulnerability, paving the way for understanding the developmental origins of mental health.

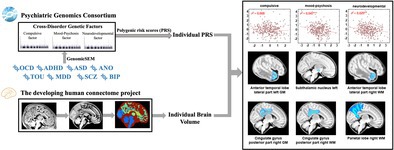

## 1 Introduction

Psychiatric disorders, including both neurodevelopmental conditions such as Attention‐Deficit/Hyperactivity Disorder (ADHD) and Autism Spectrum Disorder (ASD), as well as later‐onset illnesses like schizophrenia (SCZ) and major depressive disorder (MDD), are underpinned by complex polygenic architectures that are associated with brain phenotypes (Demontis et al. 2023; Grove et al. 2019; Guo et al. 2022; Mu et al. 2024; Trubetskoy et al. 2022; Wray et al. 2018). Large‐scale genome‐wide association study (GWAS) have identified numerous risk‐associated single‐nucleotide polymorphisms (SNPs) of psychiatric disorders, many of which are enriched in genes regulating critical neurodevelopmental processes during prenatal and early postnatal periods (Gevezova et al. 2023; Howard et al. 2019; Mullins et al. 2021). Notably, the substantial genetic overlap across psychiatric disorders suggests that early perturbations in fundamental neurodevelopmental pathways may establish vulnerability for diverse psychiatric outcomes later in life (Lee et al. 2019).

Polygenic risk scores (PRS), which aggregate these risk variants, have emerged as a powerful quantitative tool for evaluating genetic predisposition and elucidating the neurodevelopmental underpinnings of psychiatric disorders (Choi et al. 2020). Investigating the association between genetic risk profiles and neuroimaging markers provides crucial insights into how shared genetic factors relate to early brain development, thereby facilitating the identification of early risk indicators. Recent studies leveraging this approach have demonstrated that specific genetic factors significantly shape brain development during childhood (Le et al. 2023; Li et al. 2024; Morys et al. 2023; Petrican et al. 2023; Pine et al. 2023), with detectable effects even in neonatal brains (Le et al. 2023). However, most prior studies have employed disorder‐specific PRS or a single methodological approach, leaving the robustness and comparative utility of cross‐disorder PRS in the earliest phases of brain development less well characterized.

However, while disorder‐specific PRS have been extensively employed, their predictive utility for early behavioral outcomes in pediatric populations remains limited (Albiñana et al. 2023; Paul et al. 2024). This limitation likely reflects the undifferentiated nature of psychopathology in early psychopathology, wherein genetic risk manifests as transdiagnostic vulnerabilities rather than disorder‐specific manifestations (Hughes et al. 2023; Lee et al. 2019). Recent research suggests that cross‐disorder PRS, which are derived from shared genetic architecture across different psychiatric disorders, have demonstrated superior predictive performance for childhood behavioral traits compared to disorder‐ specific PRS (Hughes et al. 2023), highlighting the potential value of cross‐disorder genetic models for understanding early developmental vulnerability.

Previous studies have reported associations between psychiatric PRS and structural brain measures in pediatric populations, including a recent report of cross‐disorder PRS linked to specific brain regions in the ABCD cohort (Morys et al. 2023; Paul et al. 2024; Petrican et al. 2023; Pine et al. 2023). However, the impact of cross‐disorder genetic risk on neonatal brain development remains largely unexplored. In particular, it is unclear how these shared genetic factors influence early brain organization and whether such influences are associated with subsequent cognitive and behavioral outcomes.

**FIGURE 1:**
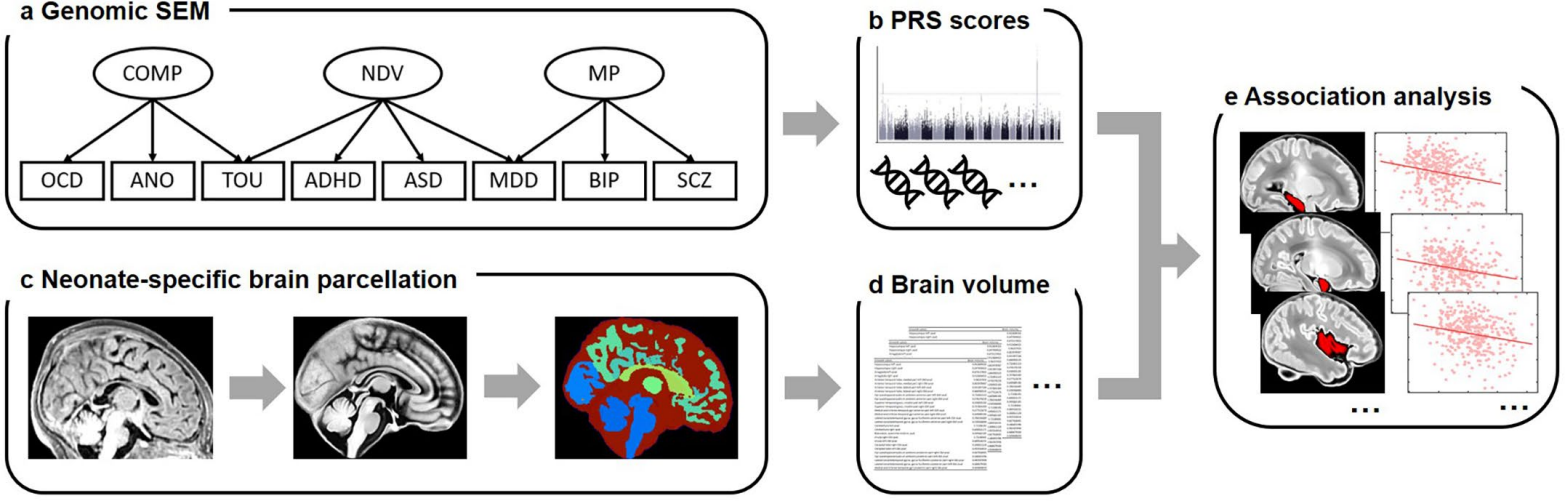
Schematic overview of the analytical workflow. (a) Derivation of three latent cross‐disorder psychiatric factors from eight major psychiatric conditions using genomic structural equation modeling (GenomicSEM). Factor loadings are provided in Table S1 (original model) and Table S2 (updated model with recent GWAS data for MDD, OCD, and BIP). (b) Calculation of polygenic risk scores (PRS) for these factors in neonates using the dHCP cohort genetic data. PRS were generated using two independent methods: PRSice‐2 (primary) and PRS‐CS (validation). (c) dHCP minimal preprocessing pipeline for structural MRI. (d) Extraction of regional brain volumes based on the neonate‐specific Draw‐EM parcellation. (e) Association analyses between PRS and brain volumes. COMP, Compulsive factor; MP, mood‐psychosis factor; NDV, Neurodevelopmental factor.

To address this gap, our study employs genomic structural equation modeling (GenomicSEM, Grotzinger et al. 2019) to derive three cross‐disorder psychiatric factors from GWAS data encompassing eight psychiatric conditions. We then calculate PRSs for these cross‐disorder factors in neonates from the Developing Human Connectome Project (dHCP) cohort. Using structural MRI data, we systematically examine and compare the associations of cross‐disorder and disorder‐specific PRS with neonatal brain volumes (Figure 1). Critically, we assess the robustness of these associations through validation analyses using updated GWAS summary statistics and an alternative PRS method (PRS‐CS). Furthermore, we evaluate how these PRS relate to early behavioral measures at 18 months of age and explore whether neonatal brain structure mediates any observed genetic‐behavioral links. By integrating genetic risk, neonatal brain structure, and early developmental outcomes within a rigorous, multi‐method framework, this work aims to uncover the neurodevelopmental pathways through which undifferentiated genetic vulnerability emerges, offering novel insights into the origins of psychiatric disorders.

## 2 Materials and Methods

### 2.1 Participants

Our study utilized data from the Developing Human Connectome Project (Edwards et al. 2022), a large‐scale perinatal brain research providing multimodal MRI scans with comprehensive collateral data including genomics, neuroimaging, and 18‐month behavioral follow‐ups collected between 2014–2022 years. From the released neonatal dataset (887 scans from 783 infants, comprising term‐born and preterm cohorts), we initially selected all available participants of European ancestry to minimize population stratification in genetic analyses. After quality control, a total of 336 neonates with both genetic and structural MRI data were included in the primary genotype–imaging analyses (173 males, 163 females). For behavioral analyses, 244 participants (125 males, 119 females) with complete genetic, neuroimaging, and 18‐month Bayley Scales of Infant Development (BSID) data were included. Written parental consent was obtained under UK Health Research Authority approval (14/LO/1169).

### 2.2 Genomic Data Acquisition and Quality Control

Saliva samples were collected from neonates and 18‐month‐old infants using Oragene DNA OG‐250 kits (DNAGenotek Inc., Canada) and genotyped on the Illumina Infinium Omni5‐4 v1.2 array, which interrogates 4,327,108 SNPs (Edwards et al. 2022). Genotyping was conducted in two batches, retaining one randomly selected sample per individual where duplicates existed. Initial quality control (QC) excluded samples with call rates < 95%, gender mismatches, or heterozygosity outliers. Subsequent SNP‐level QC removed non‐autosomal variants, those with minor allele frequency (MAF) ≤ 0.05, missingness > 2%, or significant deviations from Hardy–Weinberg equilibrium (HWE; *p* < 1e^−6^). To account for population stratification, genotypes were merged with the 1000 Genomes Phase 3 reference data (https://www.internationalgenome.org/), and population structure was assessed using principal component analysis (PCA) and uniform manifold approximation and projection (UMAP). Five major ancestry groups were identified (African, American, European, East Asian, and South Asian), and only unique European‐ancestry individuals (*n* = 471) were retained for downstream PRS analyses. Additionally, relatedness was controlled by excluding one sample from each pair with a pi‐hat ≥ 0.1875. After stringent QC, high‐quality genotype data were available for 436 infants, comprising 1,734,289 SNPs for further genetic analyses.

### 2.3 Polygenic Scores Estimation

To investigate shared genetic liability across psychiatric disorders, we applied GenomicSEM (Grotzinger et al. 2019) to derive latent factors from GWAS summary statistics of eight neuropsychiatric disorders including Attention‐Deficit/Hyperactivity Disorder (ADHD, Demontis et al. 2023), Autism Spectrum Disorder (ASD, Grove et al. 2019), Major Depressive Disorder (MDD, Wray et al. 2018), Tourette syndrome (Yu et al. 2019), Anorexia nervosa (ANO, Watson et al. 2019), Obsessive‐Compulsive Disorder (OCD, Arnold et al. 2018), Bipolar Disorder (BIP, Mullins et al. 2021), and Schizophrenia (SCZ, Trubetskoy et al. 2022). First, the GenomicSEM package was used to calculate a genetic covariance matrix (S) and its sampling covariance matrix (V) for these eight disorders using linkage disequilibrium score regression (LDSC). An exploratory factor analysis (EFA) with three factors and promax rotation was then performed on the S matrix. Consistent with the previous report (Hughes et al. 2023), this EFA yielded three clinically interpretable factors after excluding loadings less than 0.2, a neurodevelopmental factor (NDV) with primary loadings on ADHD, ASD, MDD, and Tourette syndrome; a compulsive factor (COMP) with primary loadings on anorexia nervosa, OCD, and Tourette syndrome; and a mood‐psychosis (MP) factor with primary loadings on BIP, MDD, and SCZ, accounting for approximately 60% of the genetic covariance. We then specified a confirmatory three‐factor model using the *usermodel* function in GenomicSEM to formally evaluate model fit and estimate parameters. This model demonstrated good fit to the genetic covariance data: *χ* ^2^(15) = 79.58, *p* < 0.001; Comparative Fit Index (CFI) = 0.977; Standardized Root Mean Square Residual (SRMR) = 0.069. The three latent factors were moderately correlated: neurodevelopmental factor with mood‐psychosis factor (*r* = 0.38), neurodevelopmental factor with compulsive factor (*r* = 0.24), and compulsive factor with mood‐psychosis factor (*r* = 0.46). Complete standardized factor loadings are provided in Table S1.

To test the robustness of our findings to more recent GWAS data with substantially increased sample sizes, we conducted a supplementary analysis. We replaced the summary statistics for MDD, OCD, and BIP with updated versions: MDD (Major Depressive Disorder Working Group of the Psychiatric Genomics Consortium 2025), OCD (Strom et al. 2025), and BIP (O'Connell et al. 2025). While retaining the original data for the other five disorders. The updated three‐factor model also showed good fit: *χ* ^2^(15) = 123.95, *p* < 0.001; CFI = 0.970; SRMR = 0.050. Factor correlations remained moderate (neurodevelopmental and mood‐psychosis: 0.43, neurodevelopmental and compulsive: 0.46, compulsive and mood‐psychosis: 0.48). The overall factor structure was preserved, with notable variations in specific loadings (e.g., ASD showed a secondary loading on compulsive factor in the updated model). Complete factor loadings for the updated model are provided in Table S2.

For polygenic scoring in the dHCP cohort, we computed factor‐specific polygenic risk scores (PRS) for both the original and the updated factor models. To ensure consistency with prior methodology (Hughes et al. 2023), our primary PRS construction was performed using PRSice‐2 (Choi and O'Reilly 2019). We applied clumping (LD *R* ^2^ < 0.1 within a 250 kb window) and a liberal inclusion threshold (*p* ≤ 1) to capture broad genetic effects across the genome. Variants were filtered for imputation quality (INFO ≥ 0.9), minor allele frequency (MAF ≥ 0.01), and genotype missingness (≤ 0.05). To address potential methodological considerations regarding PRS estimation (e.g., shrinkage for LD adjustment), we conducted a supplementary validation analysis using an alternative, widely‐used method: PRS‐CS (Ge et al. 2019). We applied PRS‐CS with default parameters. For both PRSice‐2 and PRS‐CS, factor‐specific PRS were generated from the respective GWAS summary statistics of the neurodevelopmental factor, compulsive factor, and mood‐psychosis factor derived from the original GenomicSEM model. All PRSs were standardized (*z*‐scored) prior to analysis.

### 2.4 Image Data Acquisition and Preprocessing

MRI scans were acquired at the Evelina Newborn Imaging Centre using a 3T Philips scanner with a dedicated neonatal imaging system during natural sleep (Hughes et al. 2017). Structural images included T1‐weighted (T1w) and T2‐weighted (T2w) sequences acquired at 0.8 × 0.8 × 1.6 mm resolution (T1w: TR = 4795 ms, TE = 8.7 ms; T2w: TR = 12,000 ms, TE = 156 ms). For structural data, we utilized the dHCP pipeline's minimally preprocessed outputs (Edwards et al. 2022), which included super‐resolution reconstruction, T1w‐T2w registration, bias correction, brain extraction, tissue segmentation, and surface reconstruction (Makropoulos et al. 2018). Subsequent volumetric analyses were performed based on the dHCP pipeline's draw‐EM neonatal‐specific brain atlas (Makropoulos et al. 2014), which parcellates the brain into 87 distinct cortical and subcortical regions. Volumetric measures for these 87 regions were extracted and served as the primary structural phenotypes in all subsequent association analyses.

### 2.5 Behavioral Questionnaires

At the 18‐month follow‐up, neurodevelopmental outcomes were assessed using the Bayley Scales of Infant and Toddler Development, Third Edition (BSID‐III), a comprehensive standardized measure of early childhood development. The BSID‐III provides evaluation across multiple domains, including cognitive, language (receptive and expressive), and motor (fine and gross motor) functions. A range of scores was utilized, including raw scores, scaled scores, composite scores, and percentile ranks for each domain. Additionally, examiner ratings during the assessment captured the child's behavioral observations across multiple dimensions, such as adaptability, alertness, and tactile responsiveness (Albers and Grieve 2007).

### 2.6 Statistical Analysis

#### 2.6.1 Polygenic Score Calculation and Association Analyses

We examined relationships between psychiatric PRS and neurodevelopmental outcomes (brain volumes and behavioral measures) using linear regression. For all analyses, standardized PRS (*z*‐scored) were regressed against each outcome variable, adjusting for gestational age, postmenstrual age at scan, sex, and the first five genetic principal components to control for population stratification. To quantify the proportion of variance in each outcome uniquely explained by the PRS, we calculated the Δ*R* ^2^ as the difference in the *R* ^2^ between a model including the PRS and all covariates and a baseline model including only the covariates.

#### 2.6.2 Brain Volume Association Analysis

We employed a multi‐step analytical framework to identify robust associations between cross‐disorder polygenic risk and neonatal brain structure. First, we performed mass‐univariate linear regressions for each of the 87 brain regions, testing associations with each cross‐disorder PRS (neurodevelopmental, compulsive, and mood‐psychosis factor) derived from our primary GenomicSEM model (using PRSice‐2). To assess the robustness of these findings, we repeated these analyses in two independent validation scenarios: (1) using PRS derived from an updated GenomicSEM model incorporating recent GWAS for MDD, OCD, and BIP, and (2) using PRS for the original factors calculated with an alternative method (PRS‐CS).

Within each analytical scenario, we applied a two‐stage approach. The first stage characterized the spatial distribution of PRS effects without including intracranial volume (ICV) as a covariate. The second stage repeated the analyses with ICV as a covariate to distinguish potential regional‐specific effects from those attributable to global brain size. Due to the substantial attenuation of statistical power after ICV adjustment, we used a nominal threshold (*p* < 0.05, uncorrected) in this second stage to identify candidate regional associations. To identify the most reliable findings, we applied a stringent triangulation criterion: a brain region was considered robustly associated with a cross‐disorder factor only if it showed a significant association (FDR‐corrected *p* < 0.05 in the first stage, or nominal *p* < 0.05 in the ICV‐adjusted stage) with that factor in all three analytical scenarios.

#### 2.6.3 Mediational Analysis of Polygenic Risk, Brain Structure, and Early Behavior

To investigate potential neurodevelopmental pathways linking genetic risk to early behavioral outcomes, we employed a two‐step analytical approach. First, we conducted linear regression analyses to identify associations between each cross‐disorder PRS and 18‐month BSID scores, adjusting for sex, gestational age, postmenstrual age at scan, and the first five genetic principal components. Given the typically small effect sizes of polygenic influences on behavioral traits, we used a lenient threshold (*p* < 0.05, uncorrected) in this initial screening. To identify the most stable associations, we repeated these analyses using PRS derived from the two validation approaches (updated GWAS data and PRS‐CS). Only behavioral measures showing a nominally significant association (*p* < 0.05) with the same PRS factor in all three analytical scenarios (primary, updated GWAS, and PRS‐CS) were carried forward to the mediation analysis.

Second, for each PRS‐behavior association that passed the above tripartite screening, we performed a mediation analysis to test whether the associated neonatal brain volume mediated the relationship. Analyses were conducted using the mediation toolbox in MATLAB, modeling the PRS as the independent variable, the associated neonatal brain volume as the mediator, and the behavioral score as the outcome, with covariates (gestational age, sex, genetic PCs) included in all paths.

### 2.7 Data Availability

The dHCP data used in this work are available online on the dHCP page (https://www.developingconnectome.org/) and access can be obtained upon application at the NDA website (https://nda.nih.gov/ccf/lifespan‐studies). The GWAS summary statistics are available following the corresponding reference; the main data come from the Psychiatric Genomics Consortium (https://pgc.unc.edu/for‐researchers/download‐results/). The genetic data of the 1000 Genomes project is available online (https://www.internationalgenome.org/).

## 3 Results

### 3.1 Distribution of Polygenic Scores and Demographic Characteristics

Following quality control, 336 European‐ancestry neonates who have both genetic and imaging data were included in the genotype‐imaging analyses (173 males/163 females; *χ* ^*2*^ = 0.298, *p* > 0.5). At the time of MRI scanning, participants had a mean postmenstrual age (PMA) of 39.73 ± 3.60 weeks and gestational age (GA) of 37.76 ± 4.11 weeks. In the primary analysis, polygenic risk scores (PRS) were calculated for the three cross‐disorder factors (Neurodevelopmental, Compulsive, and Mood‐Psychosis factors) derived from the original GenomicSEM model, as well as for eight individual disorders (ADHD, AN, ASD, BIP, MDD, OCD, SCZ, TOU), using PRSice‐2. All PRSs in this primary set were approximately normally distributed in the cohort (Jarque‐Bera test *p* > 0.05), with the exception of the disorder‐specific BIP PRS (*p* = 0.025). In validation analyses, PRS were also calculated using (1) updated GWAS summary statistics for MDD, OCD, and BIP, and (2) the alternative PRS‐CS method applied to the original factor models. The distributions of all cross‐disorder PRSs generated in these validation analyses were also approximately normal (Jarque‐Bera test *p* > 0.05). Among the updated disorder‐specific PRS, only the MDD PRS deviated from normality (*p* < 0.05).

### 3.2 Cross‐Disorder PRS Capture Broader Associations With Brain Volume Than Disorder‐Specific PRS

**FIGURE 2:**
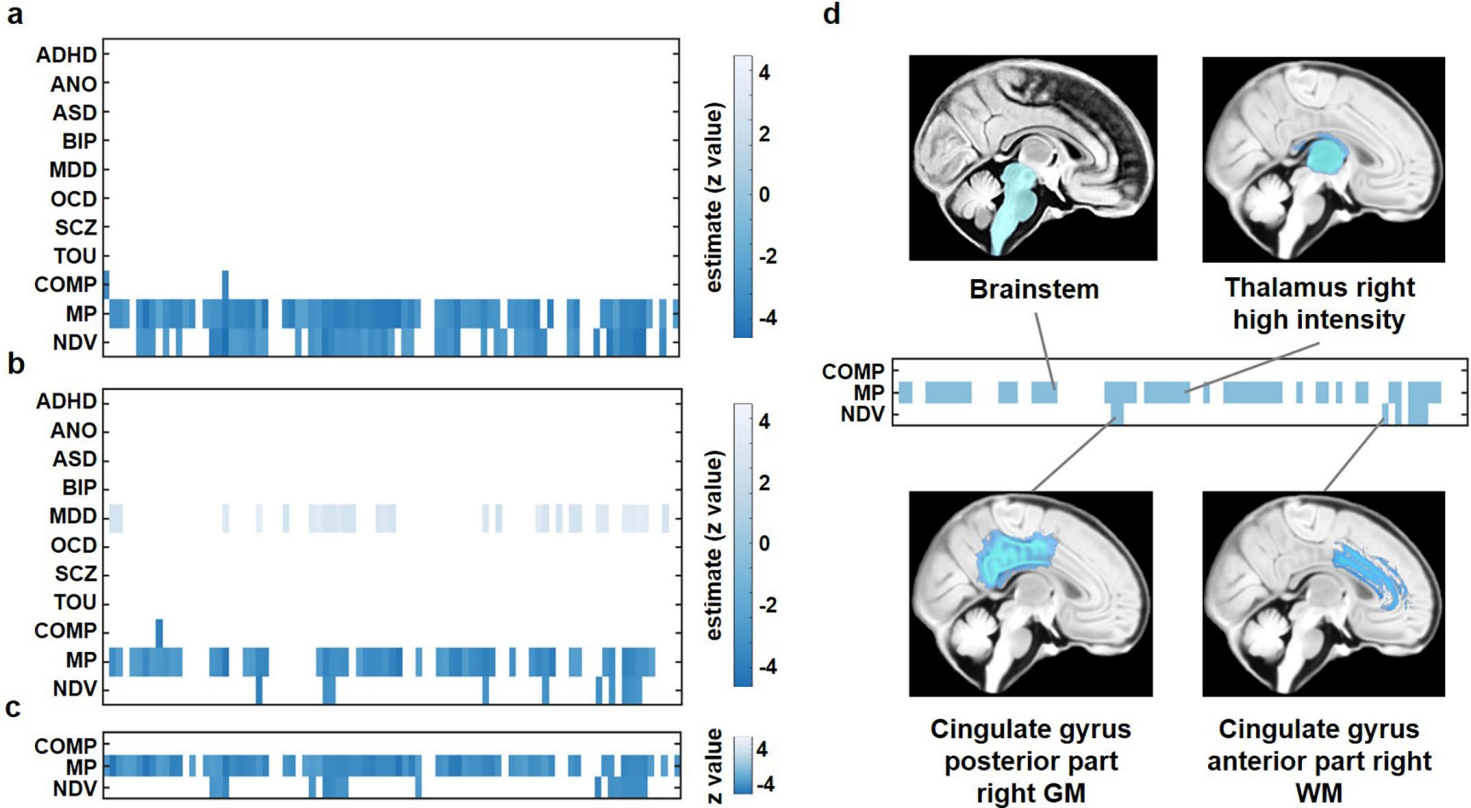
Associations between polygenic risk scores and neonatal brain volumes across primary and validation analyses. (a) heatmap showing standardized effect sizes of associations for 11 PRS with 87 neonatal brain volumes in the primary analysis. Color intensity indicates association strength; white cells denote associations that did not survive FDR correction. (b) same as (a) but for cross‐disorder PRS calculated from the GenomicSEM model fitted with updated GWAS summary statistics. (c) heatmap showing associations for the three cross‐disorder PRS calculated using the alternative PRS‐CS method. (d) matrix identifying robust associations that were statistically significant in all three analytical scenarios. The brain illustration highlights example regions that showed significant associations with the cross‐disorder PRS. COMP, compulsive factor; MP, mood‐psychosis factor; NDV, neurodevelopmental factor.

The three cross‐disorder PRSs demonstrated substantially broader associations with neonatal brain volumes compared to the eight disorder‐specific PRSs (ADHD, AN, ASD, BIP, MDD, OCD, SCZ, and TOU). Specifically, the compulsive factor PRS was significantly associated with 2 brain regions, the mood‐psychosis factor PRS with 71 regions, and the neurodevelopmental factor PRS with 52 regions (FDR corrected *p*‐value < 0.05, Figure 2a and Table S3). In contrast, none of the disorder‐specific PRSs showed any significant associations with brain volumes after multiple comparison correction. This absence of significant associations for disorder‐specific PRSs, compared to the widespread effects observed for cross‐disorder PRSs, underscores the superior capability of cross‐disorder genetic risk scores in capturing early neurodevelopmental signatures.

Validation analyses supported the robustness of these primary findings. When using updated GWAS data for MDD, OCD, and BIP, the mood‐psychosis and neurodevelopmental factors continued to show widespread significant associations (mood‐psychosis factor: 53 regions; neurodevelopmental factor: 10 regions), with substantial overlap with the primary results (mood‐psychosis factor: 51 and neurodevelopmental factor: 10 overlapping regions, respectively). The updated compulsive factor showed associations with two different regions. Notably, the MDD‐specific PRS from the updated GWAS also showed significant associations (29 regions), likely reflecting increased power from the larger sample size (Figure 2b). When using the alternative PRS‐CS method, the mood‐psychosis factor showed similarly extensive associations (71 regions), with high overlap (68 regions) with the primary analysis. The neurodevelopmental factor showed 16 significant associations with PRS‐CS, 15 of which overlapped with those identified in the primary analysis. The compulsive factor showed no significant associations (Figure 2c). Detailed validation results are provided in Tables S4 and S5.

### 3.3 Cross‐Disorder PRS Shows Differential and Widespread Associations With Neonatal Brain Volumes

To identify the most robust associations, we applied a stringent triangulation criterion: only brain regions showing significant associations (FDR *p* < 0.05) in all three analytical scenarios. The complete results from each individual analysis are presented in Figure 2 and Tables S3–S5.

Applying this strict criterion, the mood‐psychosis factor demonstrated the highest robustness, with 51 consistently associated brain regions. These included structures such as the brainstem (*β* = −0.484, SE = 0.125, *p* = 0.00013, *R* ^2^ = 3.89%), bilateral cerebellum (left: *β* = −0.437, *p* = 0.0035, R^2^ = 2.28%; right: *β* = −0.460, *p* = 0.0024, *R* ^2^ = 2.48%), and bilateral thalamus (high‐intensity part). Cortical regions with robust associations included the bilateral frontal lobes (GM), parietal lobes (GM), and their underlying white matter tracts. The proportion of variance explained (*R* ^2^) by the mood‐psychosis factor PRS for these robust regions ranged from 1.1% to 4.9%. The neurodevelopmental factor showed seven consistently associated regions. The most robust effects were observed in white matter regions, particularly the posterior cingulate gyrus (bilateral) and the frontal and parietal white matter. For example, the right parietal white matter showed *β* = −0.344 (SE = 0.084, *p* = 5.32e−05, *R* ^2^ = 4.36%). The neurodevelopmental factor PRS explained between 3.1% and 4.7% of the variance in these regions. No brain regions met the triangulation criterion for the compulsive factor, indicating that its associations in the primary analysis were not consistently replicable across different GWAS data and PRS methods (Figure 2d).

### 3.4 Regional Brain Associations With Cross‐Disorder PRS After Adjusting for Intracranial Volume

**FIGURE 3:**
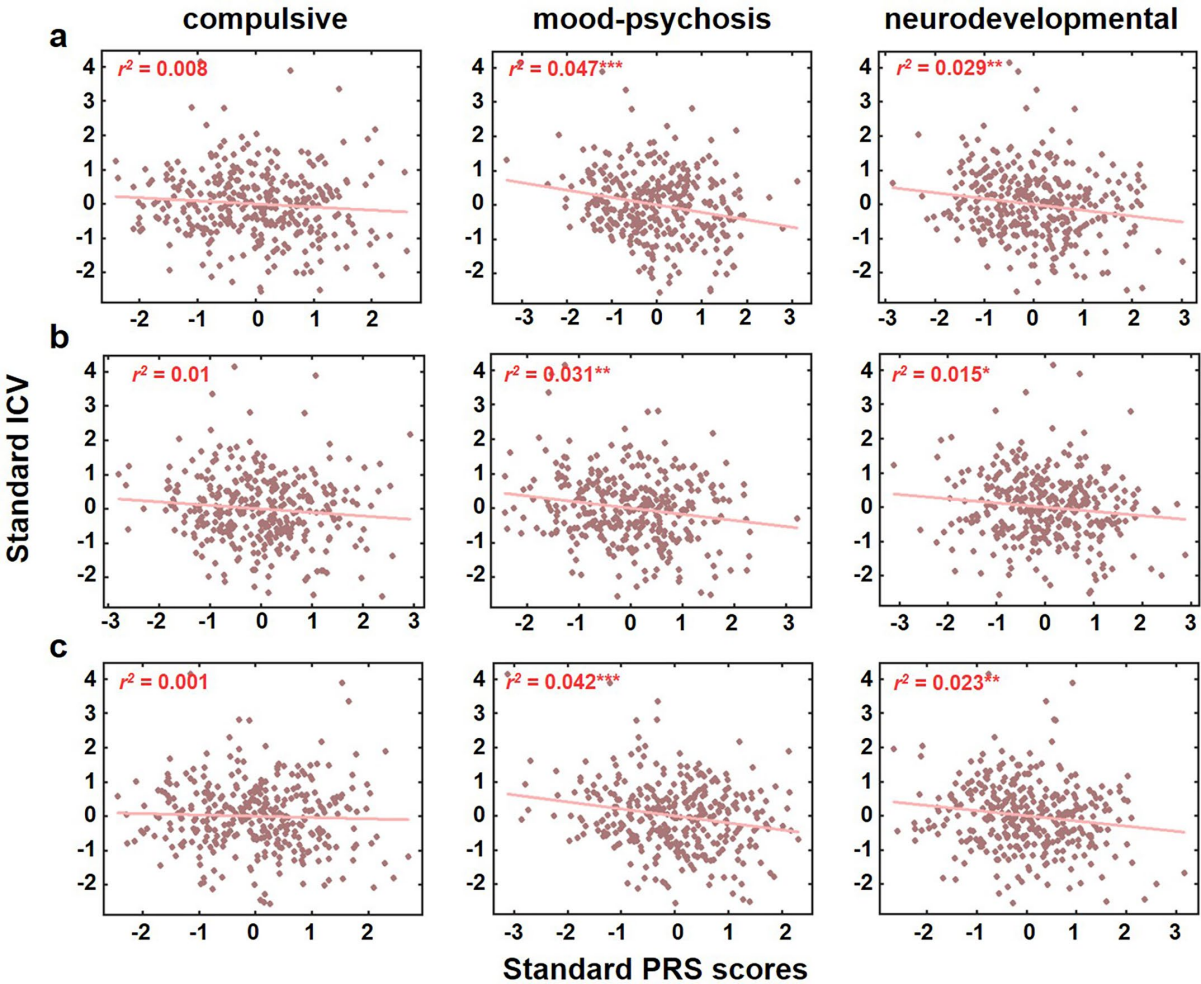
Associations between cross‐disorder polygenic risk scores and intracranial volume (ICV) across primary and validation analyses. Scatterplots illustrate the association between standardized polygenic risk scores (PRS) for the three cross‐disorder factors and standardized intracranial volume. (a) Results from the primary analysis (original GenomicSEM factors, PRSice‐2). (b) Validation analysis using factors derived from updated GWAS summary statistics (PRSice‐2). (c) Validation analysis using the alternative PRS‐CS method (original factors). Each point represents one neonate. Solid lines indicate the linear regression fit; the proportion of variance explained (*r* ^2^, *p < 0.05; **p < 0.01; ***p < 0.001) for each association are displayed within each panel.

Following the identification of widespread associations between cross‐disorder PRS and neonatal brain volumes without ICV adjustment, we next investigated whether regional‐specific effects persisted after accounting for overall brain size. We first quantified the relationship between each cross‐disorder PRS and ICV, controlling for key covariates. In the primary analysis, the mood‐psychosis factor PRS accounted for the largest proportion of variance in ICV (*R* ^2^ = 4.67%, *p* = 6.48 × 10^−5^), followed by the neurodevelopmental factor (*R* ^2^ = 2.92%, *p* = 0.0017). The compulsive factor showed a trend‐level association (*R* ^2^ = 0.82%, *p* = 0.098, Figure 3a). This pattern remained consistent in validation analyses. Using updated GWAS data, the mood‐psychosis factor explained 3.15% (*p* = 0.0011) and the neurodevelopmental factor explained 1.52% (*p* = 0.024) of the variance in ICV (Figure 3b). Similarly, with the PRS‐CS method, the mood‐psychosis factor explained 4.21% (*p* = 0.00015) and the neurodevelopmental factor explained 2.29% (*p* = 0.0055) of ICV variance. Across all analyses, the compulsive factor showed minimal association with ICV (*R* ^2^ < 1.1%, *p* > 0.05, Figure 3c). These results indicate that higher genetic risk, particularly for the mood‐psychosis factor and neurodevelopmental factor dimensions, is consistently associated with smaller intracranial volume in neonates, with the mood‐psychosis factor demonstrating the most robust and stable effect on global brain size.

The inclusion of ICV as a covariate substantially attenuated most brain volume associations. This attenuation, combined with the characteristically small effect sizes of polygenic influences—which typically require very large sample sizes (often in the thousands) for robust detection—resulted in no regional associations surviving FDR correction for any of the three cross‐disorder PRS factors. Although the dHCP cohort represents the largest currently available neonatal gene‐imaging dataset, its sample size remains underpowered to detect these subtle genetic effects after stringent multiple testing correction. Therefore, to identify potential regional effects that may inform future studies, we employed a more lenient threshold (*p* < 0.05, uncorrected) in this analysis but only focused on those brain regions that had shown significant associations in the unadjusted analysis.

**FIGURE 4:**
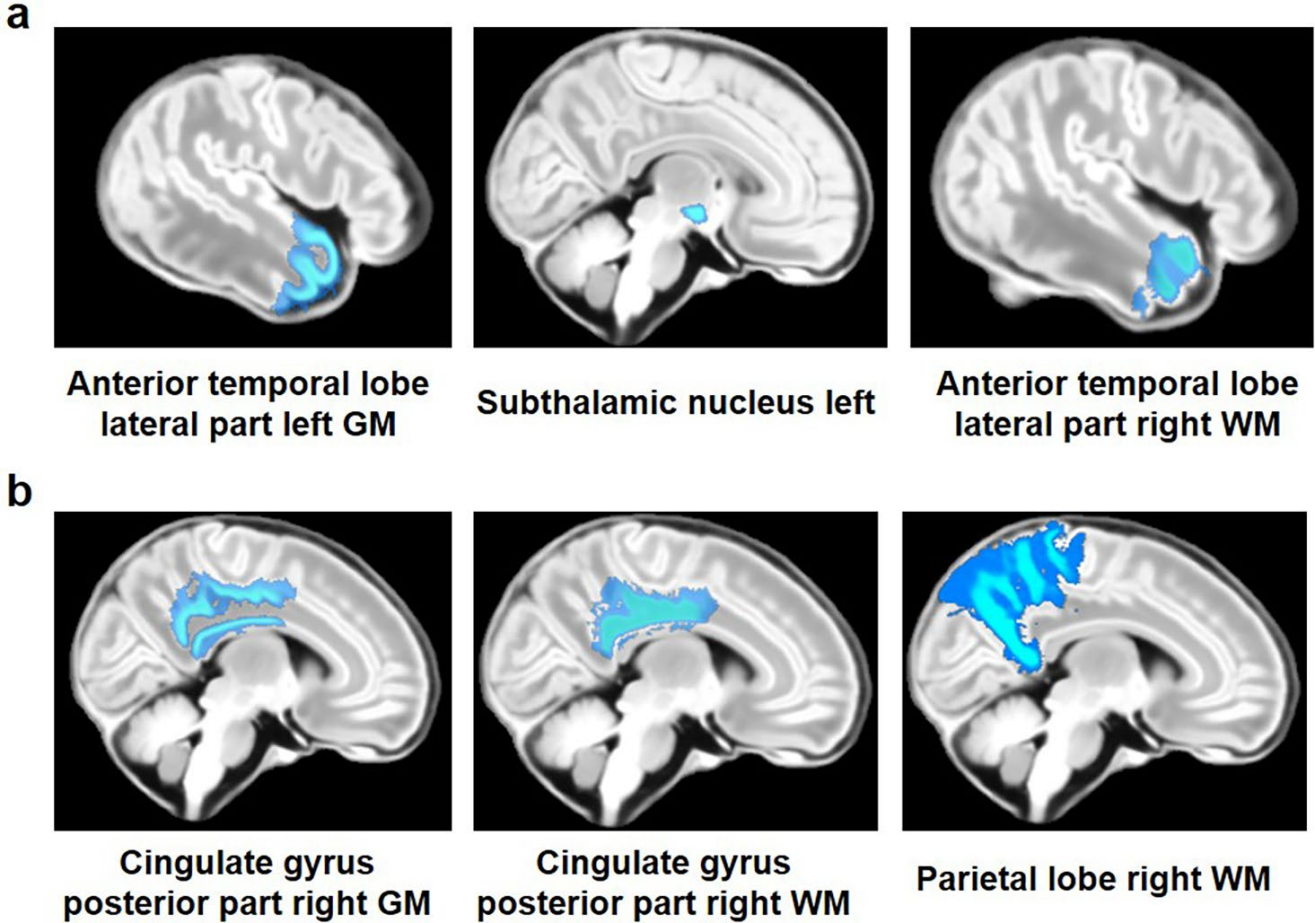
Neonatal brain regions showing nominally significant associations with cross‐disorder polygenic risk after adjustment for intracranial volume. (a) Brain regions associated with the Mood‐Psychosis factor. (b) Brain regions associated with the Neurodevelopmental factor. In both panels, brain regions are labeled according to the neonate‐specific Draw‐EM parcellation atlas. Regional names follow the convention: [Anatomical Region] [Sub‐region, if applicable] [Hemisphere: Left or Right] [Tissue Type: Gray Matter (GM) or White Matter (WM)]. All displayed associations met a nominal significance threshold in analyses adjusting for intracranial volume.

Among the 51 regions that were significantly associated with the mood‐psychosis factor in unadjusted models, three remained nominally significant after ICV adjustment (Figure 4a), including anterior temporal lobe, lateral part right GM (*β* = −0.288, SE = 0.135, *p* = 0.034, *R* ^2^ = 1.15%), left Subthalamic nucleus (*β* = −0.244, SE = 0.119, *p* = 0.041, *R* ^2^ = 1.05%), and anterior temporal lobe, lateral part right WM (*β* = −0.230, SE = 0.109, *p* = 0.035, *R* ^2^ = 1.13%).

For the neurodevelopmental factor, three of the original seven significant regions retained nominal significance after ICV adjustment (Figure 4b), including cingulate gyrus, posterior part right GM (*β* = −0.316, SE = 0.022, *p* = 0.022, *R* ^2^ = 1.36%), cingulate gyrus, posterior part right WM (*β* = −0.296, SE = 0.120, *p* = 0.014, *R* ^2^ = 1.58%), and parietal lobe right WM (*β* = −0.329, SE = 0.140, *p* = 0.019, *R* ^2^ = 1.44%). The complete set of association statistics for all brain regions is provided in Table S6.

### 3.5 Associations Between Cross‐Disorder PRS and Early Childhood Behaviors

We examined the relationships between cross‐disorder PRS and early neurodevelopmental outcomes in 244 neonates with complete genetic, neuroimaging, and behavioral data (125 males, 119 females; *χ* ^2^ = 0.15, *p* > 0.7). Behavioral assessments using the Bayley Scales of Infant Development (BSID‐III) were conducted at a mean age of 19.49 ± 2.45 months. The sample had a mean gestational age of 38.28 ± 3.84 weeks.

In the primary analysis, linear regressions adjusting for sex, scan age, gestational age, and genetic PCs revealed several nominally significant associations (*p* < 0.05, uncorrected). The compulsive factor PRS was positively associated with motor skills (composite score: *β* = 0.129, SE = 0.065, *p* = 0.047, *R* ^2^ = 1.60%; percentile score: *β* = 0.135, SE = 0.065, *p* = 0.038, *R* ^2^ = 1.75%), tactile responsiveness (*β* = 0.143, SE = 0.064, *p* = 0.026, *R* ^2^ = 2.02%), and alertness (*β* = 0.128, SE = 0.064, *p* = 0.045, *R* ^2^ = 1.63%). The mood‐psychosis factor PRS was positively associated with tactile responsiveness (*β* = 0.168, SE = 0.063, *p* = 0.008, *R* ^2^ = 2.80%). The neurodevelopmental factor PRS showed positive associations with engagement score (*β* = 0.133, SE = 0.063, *p* = 0.023, *R* ^2^ = 1.75%), alertness (*β* = 0.200, SE = 0.062, *p* = 0.001, *R* ^2^ = 3.94%), and a negative association with adaptability (*β* = −0.142, SE = 0.062, *p* = 0.023, *R* ^2^ = 2.03%, Table S7).

**FIGURE 5:**
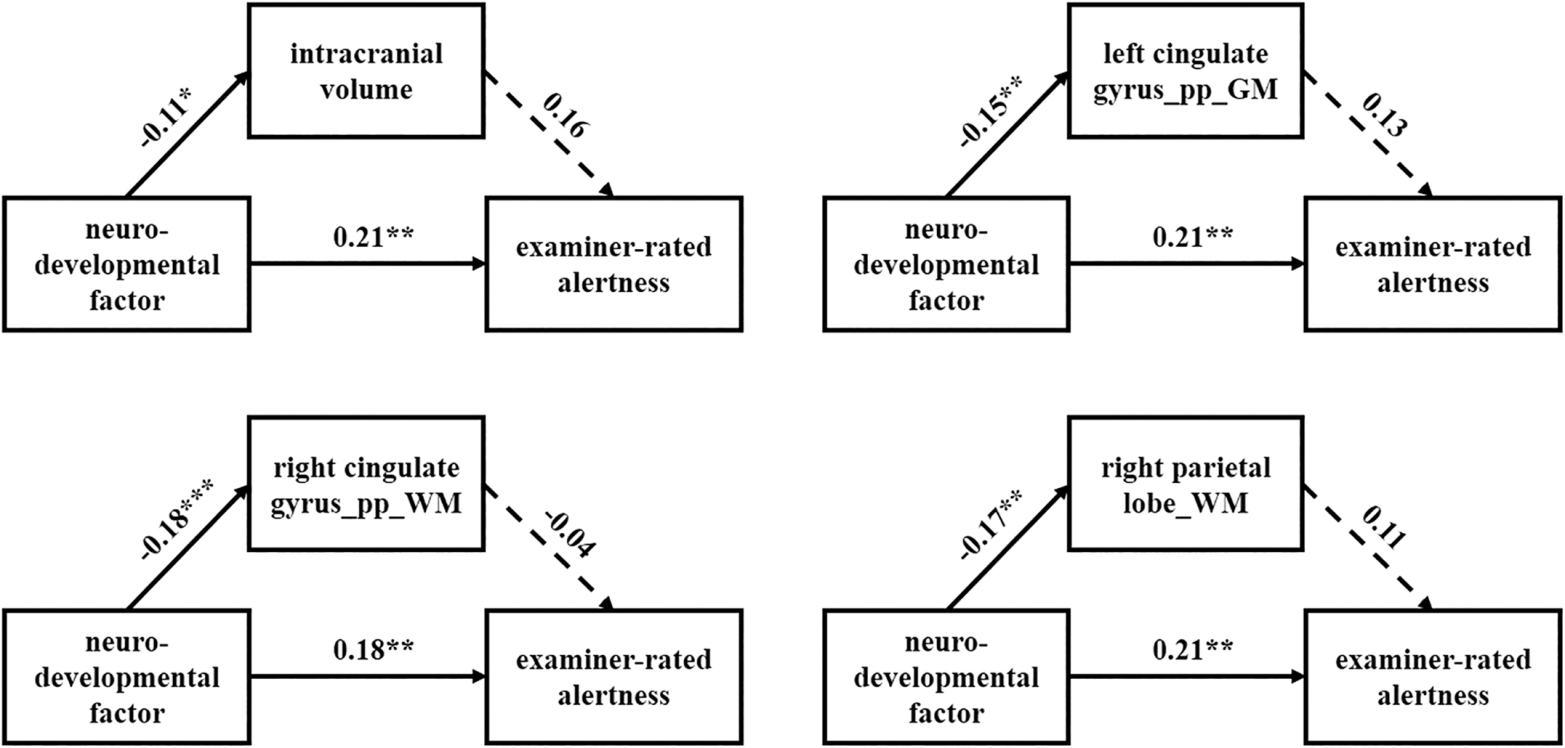
Mediation analysis testing neonatal brain measures as mediators between neurodevelopmental (NDV) polygenic risk and alertness at 18 months. Each panel shows path coefficients for one of four candidate brain measures: Intracranial volume and three NDV‐associated regional volumes. Solid lines represent significant paths (*β*, *p < 0.05; **p < 0.01; ***p < 0.001), dashed lines represent non‐significant associations.

To identify robust associations, we applied a stringent criterion requiring nominal significance (*p* < 0.05) in all three analytical scenarios (primary, updated GWAS, and PRS‐CS). Only one association met this stability criterion: the positive relationship between the neurodevelopmental factor PRS and examiner‐rated alertness (Table S7). We proceeded to test whether this robust association was mediated by neonatal brain structure. Four candidate brain measures were examined as potential mediators: intracranial volume (ICV) and three regional volumes that were consistently and significantly associated with the neurodevelopmental factor across all analytical scenarios. The neurodevelopmental factor PRS had a significant total effect on alertness (c path *β* = 0.206, *p* = 0.0025). However, none of the mediation analyses yielded a significant indirect effect (all ab path *p* > 0.05). The direct effect of neurodevelopmental factor PRS on alertness remained significant after accounting for each brain measure (c′ path *p*‐values range: 0.0027–0.0092). This pattern suggests that while neurodevelopmental factor genetic risk directly influences both brain structure and behavior, the tested brain volumes do not statistically mediate the pathway to early alertness. The lack of significant mediation likely stems from the limited association between these specific brain volumes and the behavioral outcome (b paths were non‐significant, *p* > 0.05, Figure 5).

## 4 Discussion

In this study, we investigated the associations between cross‐disorder polygenic risk and neonatal brain structure, leveraging latent psychiatric factors derived from GenomicSEM. Our principal findings reveal that polygenic risk for cross‐disorder psychiatric dimensions, particularly the neurodevelopmental and mood‐psychosis factors, is robustly associated with smaller intracranial volume (ICV) in neonates. This global brain size effect accounted for a substantial portion of the observed associations with regional volumes. When this global effect was statistically controlled, a subset of regions, most consistently identified across validation analyses, continued to show associations. In contrast, disorder‐specific PRS showed no significant associations after multiple comparison correction. Exploratory behavioral analyses identified a single robust link between neurodevelopmental factor polygenic risk and heightened alertness at 18 months. In addition, these main results were observed when we repeated the analyses using updated GWAS summary statistics and an alternative PRS construction approach, supporting the robustness of our results.

A key finding of our work is the superior capability of cross‐disorder PRS over disorder‐specific PRS in capturing early neurodevelopmental signatures. This result aligns with previous literature suggesting that the genetic underpinnings of psychopathology are pleiotropic and transdiagnostic, especially during early development (Hughes et al. 2023; Lee et al. 2019). The undifferentiated nature of genetic risk in the neonatal period may manifest as broad alterations in brain structure, which only later, through complex gene–environment interactions and developmental trajectories, canalize into specific psychiatric syndromes. Our findings, strengthened by rigorous validation, are consistent with previous work in pediatric populations reporting that cross‐disorder PRS are more strongly predictive of brain morphology and behavioral traits than disorder‐specific scores (Hughes et al. 2023). This underscores the advantage of using cross‐disorder genetic constructs to elucidate the shared neurodevelopmental origins of psychiatric vulnerability (Hughes et al. 2023; Lee et al. 2019; Paul et al. 2024).

The inclusion of ICV as a covariate in our models led to a substantial attenuation of statistical significance for most brain regions. This attenuation critically delineates the nature of the observed associations: a substantial portion of the polygenic risk captured by the neurodevelopmental and mood‐psychosis factors is related to global brain size. This widespread effect on overall brain volume is consistent with the fundamental role of shared genetic factors in regulating general neurodevelopmental processes, such as neurogenesis and synaptogenesis (Lee et al. 2019). Our findings therefore suggest that a primary, detectable manifestation of the shared genetic liability for major psychiatric disorders in the neonatal period is a subtle but widespread association with early brain growth.

Although most associations reflected global brain size differences, our exploratory analysis using a lenient threshold (*p* < 0.05, uncorrected) highlighted several regions where associations with genetic risk persisted after accounting for ICV, warranting cautious interpretation. Each of the robust cross‐disorder factors was linked to a distinct pattern in these exploratory analyses. For instance, the mood‐psychosis factor was nominally associated with regions including the anterior temporal lobe (lateral part, right GM and WM) and the left subthalamic nucleus. This pattern is of interest as the temporal lobe is consistently implicated in SCZ and BIP and involved in memory and emotional processing (Jones et al. 2009; Wei et al. 2025; Zhang et al. 2018). For the neurodevelopmental factor, nominal associations were observed in regions such as the posterior cingulate gyrus (right GM and WM) and the right parietal white matter. This regional pattern aligns with theories of altered structural connectivity in neurodevelopmental conditions like ADHD (Parlatini et al. 2023) and ASD (Zhang et al. 2022). These exploratory, ICV‐adjusted findings highlight specific neuroanatomical candidates that may be differentially linked to distinct genetic risk dimensions, beyond their shared association with global brain size.

Finally, we conducted exploratory analyses to investigate whether the cross‐disorder genetic risk dimensions were associated with observable behavioral dimensions as early as 18 months, and to preliminarily assess whether neonatal brain structure might mediate such links. After applying a stringent criterion requiring nominal significance across all three analytical scenarios (primary, updated GWAS, and PRS‐CS), only one association demonstrated stability: a positive relationship between the neurodevelopmental factor PRS and examiner‐rated alertness. Follow‐up mediation analyses did not yield significant indirect effects through intracranial volume or the neurodevelopmental factor‐associated regional brain volumes, suggesting that the observed genetic‐behavioral link for alertness was not statistically mediated by these specific neonatal brain structural measures. The general absence of strong, statistically robust associations between the PRS and most BSID measures is not unexpected, given the characteristically small effect sizes of polygenic influences, the limited sample size for behavioral analyses, and the fact that complex behavioral phenotypes are likely less genetically constrained in infancy than brain structure. However, the replicable link between the neurodevelopmental genetic risk dimension and heightened alertness provides a specific, albeit preliminary, clue for future research. This finding aligns with suggestions that altered arousal regulation may be an early marker of regulatory difficulties often preceding later neurodevelopmental diagnoses (Abramov et al. 2019; Burns and Martin 2014; Lee and Bo 2021). Future longitudinal studies with larger cohorts are needed to trace how this and other early behavioral signatures, potentially in interaction with later environmental exposures, evolve into overt clinical symptoms.

## 5 Limitation

Several limitations of this study should be acknowledged. The modest sample size, while comparable to other neonatal imaging cohorts, constrained our statistical power to detect subtle polygenic effects, particularly for behavioral analyses and after stringent multiple comparison corrections or adjustment for global brain size (ICV). Consequently, regional associations identified after ICV adjustment must be considered exploratory and require replication. The polygenic scores were constructed based on GWAS summary statistics from adult populations, which may not fully capture developmental‐stage‐specific genetic influences on the neonatal brain. Additionally, both the GWAS summary statistics and our target neonatal cohort are primarily of European ancestry. This limits the generalizability of our findings, and future studies with large, diverse cohorts are needed to obtain more universally applicable conclusions. Furthermore, our participants were drawn from a general population cohort without clinical diagnoses, limiting direct translation to psychiatric outcomes. While standardized, the Bayley Scales may lack the precision of more direct neurophysiological measures and are subject to developmental instability characteristic of infancy. Finally, our study design is associative, and the nature of PRS analysis precludes causal inference regarding the relationship between genetic liability and brain structure. Future studies with larger, longitudinally followed clinical cohorts will be essential for validation.

## 6 Conclusion

This study demonstrates that cross‐disorder polygenic risk scores capture distinct spatial patterns of association with neonatal brain structure, providing evidence for the earliest neurodevelopmental correlates of psychiatric genetic vulnerability. Cross‐disorder PRS, particularly the neurodevelopmental and mood‐psychosis factors, showed substantially broader associations with brain volumes than disorder‐specific PRS. The most robust finding was a shared association of the neurodevelopmental factor and mood‐psychosis factor genetic liability with smaller global brain size. Exploratory analyses, after accounting for intracranial volume, provided tentative hints of additional region‐specific patterns linked to distinct genetic risk dimensions. Furthermore, exploratory behavioral analyses identified a specific link between the neurodevelopmental factor and heightened alertness at 18 months. Collectively, these findings indicate that genetic liabilities for psychiatric disorders are associated with variations in brain development at birth, primarily through a global effect, with suggestive evidence for more specific neuroanatomical and behavioral correlates.

## Funding

This work was supported by the National Natural Science Foundation of China (32400887), the Zhejiang Provincial Natural Science Foundation of China (LQ23C090008), the Zhejiang Provincial Philosophy and Social Science Planning Project: Provincial‐Municipal Collaboration (25SSHZ006YB), and the Ministry of Science and Technology of the People's Republic of China (2021ZD0200202).

## Disclosure

My article reports human subjects. Recruitment meets scientific requirements & HBMs expectation of inclusivity.

## Conflicts of Interest

Associate Editor is co‐author—Dan Wu is a handling editor of <Human Brain Mapping> and a co‐author of this article. To minimize bias, they were excluded from all editorial decision‐making related to the acceptance of this article for publication.

## Supporting information


**Table S1:** Exploratory Factor Analysis Results of the Original Three‐Factor GenomicSEM Model (Based on Hughes et al. 2023 Framework).
**Table S2:** Exploratory Factor Analysis Results of the Updated GenomicSEM Model (Incorporating Recent GWAS for MDD, OCD, and BIP).
**Table S3:** Primary analysis: Associations between cross‐disorder PRS (original GWAS, PRSice‐2) and neonatal brain volumes.
**Table S4:** Validation analysis: Associations between cross‐disorder PRS (updated GWAS, PRSice‐2) and neonatal brain volumes.
**Table S5:** Method validation: Associations between cross‐disorder PRS (original GWAS, PRS‐CS) and neonatal brain volumes.
**Table S6:** Associations between cross‐disorder polygenic risk scores and neonatal brain volumes after adjusting for intracranial volume (ICV)
**Table S7:** Associations Between Comorbid Polygenic Risk Scores and 18‐Month Behavioral Outcomes in Primary and Validation Analyses.

## Data Availability

The data that support the findings of this study are available from The developing human connectome project. Restrictions apply to the availability of these data, which were used under license for this study. Data are available from https://www.developingconnectome.org/ with the permission of The developing human connectome project.
